# DNA repair protein APE1 is involved in host response during pneumococcal meningitis and its expression can be modulated by vitamin B6

**DOI:** 10.1186/s12974-017-1020-5

**Published:** 2017-12-12

**Authors:** Leonam G. Coutinho, Ana Helena Sales de Oliveira, Matthias Witwer, Stephen L. Leib, Lucymara F. Agnez-Lima

**Affiliations:** 1 Departamento de Biologia Celular e Genética, Centro de Biociências, Universidade Federal do Rio Grande do Norte, UFRN, Campus Universitário, Lagoa Nova, Natal, RN 59078-900 Brazil; 2 Departamento de Bioquímica, Instituto de Química, Universidade de São Paulo, USP, São Paulo, Brazil; 3 Institute for Infectious Diseases, University of Bern, Friedbuehlstrasse 51, CH-3010 Bern, Switzerland; 4 Instituto Federal de Educação Tecnológica do Rio Grande do Norte, IFRN, Natal, Brazil

**Keywords:** APE1, Pneumococcal meningitis, Vitamin B6, Cortex, Hippocampus, Oxidative stress

## Abstract

**Background:**

The production of reactive oxygen species (ROS) during pneumococcal meningitis (PM) leads to severe DNA damage in the neurons and is the major cause of cell death during infection. Hence, the use of antioxidants as adjuvant therapy has been investigated. Previous studies have demonstrated the possible participation of apurinic/apyrimidinic endonuclease (APE1) during PM. The aims of this study were to investigate the APE1 expression in the cortical and hippocampal tissues of infant Wistar rats infected with *Streptococcus pneumoniae* and its association with cell death and understand the role of vitamin B6 (vitB6) as a protective factor against cell death.

**Methods:**

APE1 expression and oxidative stress markers were analyzed at two-time points, 20 and 24 h post infection (p.i.), in the cortex (CX) and hippocampus (HC) of rats supplemented with vitB6. Statistical analyses were performed by the nonparametric Kruskal–Wallis test using Dunn’s post test.

**Results:**

Our results showed high protein levels of APE1 in CX and HC of infected rats. In the CX, at 20 h p.i., vitB6 supplementation led to the reduction of expression of APE1 and apoptosis-inducing factor, while no significant changes in the transcript levels of caspase-3 were observed. Furthermore, levels of carbonyl content and glutamate in the CX were reduced by vitB6 supplementation at the same time point of 20 h p.i.. Since our data showed a significant effect of vitB6 on the CX at 20 h p.i. rather than that at 24 h p.i., we evaluated the effect of administering a second dose of vitB6 at 18 h p.i. and sacrifice at 24 h p.i.. Reduction in the oxidative stress and APE1 levels were observed, although the latter was not significant. Although the levels of APE1 was not significantly changed in the HC with vitB6 adjuvant therapy, vitB6 supplementation prevented the formation of the truncated form of APE1 (34 kDa) that is associated with apoptosis.

**Conclusions:**

Our data suggest that PM affects APE1 expression, which can be modulated by vitB6. Additionally, vitB6 contributes to the reduction of glutamate and ROS levels. Besides the potential to reduce cell death and oxidative stress during neuroinflammation, vitB6 showed enhanced effect on the CX than on the HC during PM.

## Background

Pneumococcal meningitis (PM) is one of the most common and serious infections of the central nervous system (CNS). Despite the efficacy of antibiotic therapy, cellular injuries and neurological sequelae are still common. Cortical necrosis and hippocampal apoptosis are associated to PM in human and animal models. Over the last decades, studies have shown that reactive oxygen species (ROS) play a central role in brain damage during bacterial meningitis and treatment with various radical scavengers/antioxidants inhibited neuronal loss [1–3]. Oxidative stress is one of the main causes of DNA damage resulting in base modifications and single- and double-strand breaks. The base excision repair (BER) pathway is involved in the repair of oxidized DNA lesions in neurons and has been associated with several neurodegenerative diseases [4, 5]. The apurinic/apyrimidinic endonuclease 1 (APE1) acts in the BER pathway upon removal of damaged bases by glycosylases [6, 7]. In addition, APE1 is a multifunctional protein related to DNA repair and regulation of gene expression and therefore has emerged as an important factor in immune and inflammatory responses [8].

APE1 is highly expressed in some regions of the central nervous system and promotes survival of neurons after oxidative stress [4, 5]. Cerebral ischemia and reperfusion induce accumulation of oxidative DNA lesions in the brain, which, if not promptly repaired, may trigger cell death. Conversely, reduction in APE1 expression was observed in the hippocampus following hypoxic–ischemic injury [9], in the cortex after compression injury [10] and in the spinal cord after ischemia [11]. The decrease in APE/Ref-1 expression was also associated with cortical infarction after photothrombotic cerebral ischemia [12]. In the caudal region of the spinal cord, a strong correlation between DNA damage and the increase in APE1 mRNA levels was observed after traumatic spinal cord injury [13]. However, APE1 expression in brain tissues has been poorly studied during infectious disease, although some data have shown association of APE1 polymorphism with the occurrence of bacterial meningitis [14, 15].

Previously, in vivo experiments showed that supplementation of vitamin B6 (vitB6) during experimental PM improved the cell survival by reducing hippocampal apoptosis through maintenance of cellular energy stores [16]. VitB6 is a hydro-soluble molecule naturally found in several foods and available as a dietary supplement. It is estimated that it performs around 4% of catalytic activity in the human system as a coenzyme mostly involved in the protein synthesis. Additionally, vitB6 contributes to a wide range of functions in human metabolic processes of carbohydrates and lipids and plays an important role in the cognitive development by biosynthesis of neurotransmitters [17]. Epidemiological studies indicate that vitB6 has beneficial effects on human health by protecting against several pathologies such as cancer, diabetes, cardiovascular disease, and neurological disorders [18]. The vitamin deficiency is associated with immune system impairments such as reduction in the numbers and functions of immunological cells [19], and intake of vitB6 is linked to the protection against inflammation [20].

Overall, since multiple literature evidences show APE1 as an important enzyme during oxidative stress conditions in the brain, we focused on investigating its expression in the cortex (CX) and hippocampus (HC) during experimental PM after vitB6 treatment which may contribute to the understanding of the role for this vitamin as an adjuvant therapy to reduce oxidative stress during neuroinflammatory diseases.

## Methods

### Infant rat model of pneumococcal meningitis

Animal studies were approved by the Animal Care and Experimentation Committee of Canton of Bern, Switzerland, and followed the guidelines of the National Institutes of Health for performing animal experiments. Wistar rats were infected intracisternally on postnatal day 11 with 10 μL of saline containing *S. pneumoniae* (7 × 10^5^ cfu/mL), with a 32-gauge needle. Infected animals were randomly divided in two groups: the first group received the treatment with vitB6 subcutaneously (s.c.; 600 mg/kg, 0 h post infection (p.i.)), while the second group received an equal volume (360 μL) of saline. Uninfected control animals were injected with 10 μL of sterile saline solution in the cisterna magna. Cerebrospinal fluid (CSF) was obtained by puncture of the cisterna magna at 18 h p.i., and 5 μL was cultured qualitatively to document meningitis. Infected animals were confirmed by positive bacterial titers in the CSF (log10 6.4–log10 6.8 cfu/mL) and clinical signs according to the following score system: 1 for comatose animals; 2 for rats that do not turn upright after positioning on the back; 3 for animals that turn within 30 s; 4 for animals that turn upright less than 5 s; and 5 for rats with normal activity [2]. Despite the vitB6 treatment, the infected animals did not show a significant difference about CSF bacterial titers, weight, and clinical course of meningitis. All animals received antibiotic therapy at 18 h p.i. (ceftriaxone, 100 mg/kg subcutaneous injection; Roche Pharma, Reinach, Switzerland). Animals were sacrificed at predetermined time points, 20 h (in total *n* = 19) and 24 h (in total *n* = 20) p.i. by an overdose of intraperitoneally administered pentobarbital (100 mg/kg). These chosen time points have been associated with high score of apoptosis during PM.

Another group of infected rats (*n* = 12) was subjected to similar treatment as previously described; however, six animals received an extra dose of vitB6 supplementation at the same time point as antibiotic therapy, while the others (*n* = 6) received the same volume of saline, and were sacrificed at 24 h p.i.. The animals that received vitB6 at the time of infection were named “PM/pre sup vitB6,” while the animals supplemented with the second dose of vitB6 were named “PM/pre pos sup vitB6” and its control PM2. Since no statistical differences between PM and PM2 were observed, these animals were only named as PM.

In resume, the animals evaluated in this work were distributed into the following groups: uninfected control animals (CTRL) 20 h (*n* = 5), uninfected control animals (CTRL) 24 h (*n* = 5), infected animals (PM) without vitB6 treatment 20 h p.i. (*n* = 5), infected animals (PM) without vitB6 treatment 24 h p.i. (*n* = 5), infected animals plus vitB6 treatment (PM/pre sup vitB6) 20 h p.i. (*n* = 9), infected animals plus vitB6 treatment (PM/pre sup vitB6) 24 h p.i (*n* = 10), infected animals without the second dose of vitB6 (PM) 24 h (*n* = 6), and infected animals plus the second dose of vitB6 treatment (PM/pre pos sup vitB6) 24 h p.i. (*n* = 6). These animals were used in all assays described below.

After perfusion with ice-cold phosphate buffer saline (PBS), the brains were dissected, rolled on a filter paper to remove the meninges, and cut into two hemispheres, and the CX and HC were isolated in ice cold PBS from both hemispheres. The right half of the brains were frozen on dry ice and stored at − 80 °C for protein extraction and western blot, carbonyl content, and glutamate level assays, while the left half was kept in RNAlater (Ambion Europe Ltd., Huntingdon, Cambridgeshire, UK) in PBS (*v*/*v* 1:2) at 4 °C for RNA extraction.

### RNA isolation and RT-qPCR

Total RNA was extracted from the tissue samples of CX and HC from animals using RNeasy® Lipid Tissue kit (QIAGEN, Basel, Switzerland) and purified with RNeasy columns (QIAGEN, Basel, Switzerland). The quantification and assessment of RNA integrity were performed on the Agilent 2100 bioanalyzer platform (RNA 6000 Nano, Agilent technologies, Waldbronn, Germany) and validated on the NanoDrop® (NanoDrop, Wilmington, USA) quantification device. The OmniScript First Strand cDNA Synthesis System (Qiagen) was used with 2 μg total RNA and oligo-dT primers (Promega, Madison, WI, USA), following the manufacturer’s protocol. To minimize the methodological effects interfering with cDNA quantification, all total RNA samples were processed simultaneously. Template cDNA was amplified using the Step One Detection system (Applied Biosystems, Foster, CA, USA). SYBR-green PCR Master Mix (Qiagen, Basel, Switzerland) was used for the amplification and detection of APE1, apoptosis-inducing factor (AIF), caspase-3 (Casp-3), and glyceraldehyde-3-phosphate dehydrogenase (GAPDH) transcripts. The specificity of the SYBER-green signal was validated by the presence of single and sharp melting peak for every PCR run. The amount of template used for the PCR reaction was normalized to the commonly used housekeeper gene GAPDH. APE1 oligonucleotide primers used in this study were designed with Primer 3 with the following sequences: forward 5′ *TGCTCCAGACGCCTAAGGGCTTT* 3′ and reverse 5′ *TCTGGCTCGGACTTGGGTTCTTCC* 3′, while the primers for AIF (RTPrimerDB ID: 3945), Casp-3 (RTPrimerDB ID: 1207), and GAPDH (RTPrimerDB ID: 192) were obtained from the public database RTprimerDB.

### Protein extraction and western blot

The frozen HC and CX samples were homogenized in 1:3 (*w*/*v*) and 1:5 (*w*/*v*) ratios, respectively, in ice-cold homogenization buffer (50 mM Tris, 2 mM MgCl_2_ at pH 8.0, 0.4 mM PMSF, 0.4 mM NaCl, 1% NP-40) with TissueRuptor, a rotor-stator homogenizer (Qiagen, Basel, CH). The homogenates were centrifuged (10,000×*g*, 15 min, 4 °C), supernatant transferred, aliquoted, and frozen on dry ice. The protein concentration in the supernatant was measured using Bradford assay. For assessing APE1 levels, 10 μg of total protein of hippocampal or cortical protein extracts was separated on 12% SDS-PAGE and transferred onto a PVDF membrane. The membrane was blocked for 1 h in PBS containing 5% milk and 0.01% Tween 20 and incubated overnight with monoclonal mouse anti-APE1 (1:1000; Santa Cruz Biotechnology, Santa Cruz, CA, USA) and anti-β actin (1:1000; Santa Cruz Biotechnology, Santa Cruz, CA, USA) antibodies. The blots were washed with PBST and incubated with the secondary antibody HRP conjugate (1:1000; Santa Cruz Biotechnology, Santa Cruz, CA, USA) for 1 h, exposed to ECL Prime western blotting detection reagent (Amersham), and analyzed by Chemidoc (Biorad).

### Glutamate determination

Glutamate levels were determined using the glutamate assay kit (BioVision, CA, USA). Briefly, total protein extract from both tissues was homogenized with the provided assay buffer and mixed with the glutamate developer and enzyme mix. Next, the total reaction was incubated for 30 min at 37 °C in dark, followed by measurement of samples at 450 nm in a microplate reader (MicroQuant, Biotek).

### Carbonyl content

The carbonyl content was determined spectrophotometrically using the 2,4-dinitrophenylhydrazine (DNPH)-labeling. Briefly, a set of protein samples from animals, with a final volume of 200 μL (1 mg/mL), was incubated for 1 h with 200 μL DNPH in the dark, while another set of the same samples was incubated with 200 μL of HCl for 1 h. All samples were sequentially extracted with 50% (*w*/*v*) trichloroacetic acid at − 20 °C for 1 h and washed three times with ethanol/ethyl acetate 1:1 (*v*/*v*). The resulting precipitate was dissolved in 500 μL of 6 M guanidine hydrochloride, and the samples were measured at 370 nm in a microplate reader (MicroQuant, Biotek). Difference spectrum of the samples treated with DNPH versus similar samples treated with HCl was used as the corrected absorbance (CA). Final results were obtained from the following calculation: protein carbonyl (nmol/mL) = [(CA)/0.011 μM^−1^] (500 μL/200 μL).

### Statistical analysis

Samples were tested for normal distribution using D’Agostino & Pearson omnibus normality test (GraphPad Prism, version 4.0, GraphPad Software Inc., San Diego, CA, USA). The comparisons among three or more unmatched groups were performed by the nonparametric Kruskal–Wallis test while differences between two groups were tested using Dunn’s post test (GraphPad Prism, version 4.0, GraphPad Software Inc., San Diego, CA, USA). A value of *p* < 0.05 was considered statistically significant.

## Results

### Establishing experimental pneumococcal meningitis

All infected animals developed meningitis as evidenced by the positive bacterial CSF titers at 18 h p.i.. Within this time period, clinical signs of meningitis were documented in all the infected animals, but not in control animals (data not shown).

### Analysis of transcript expression in cortex and hippocampus during pneumococcal meningitis

**Fig. 1:**
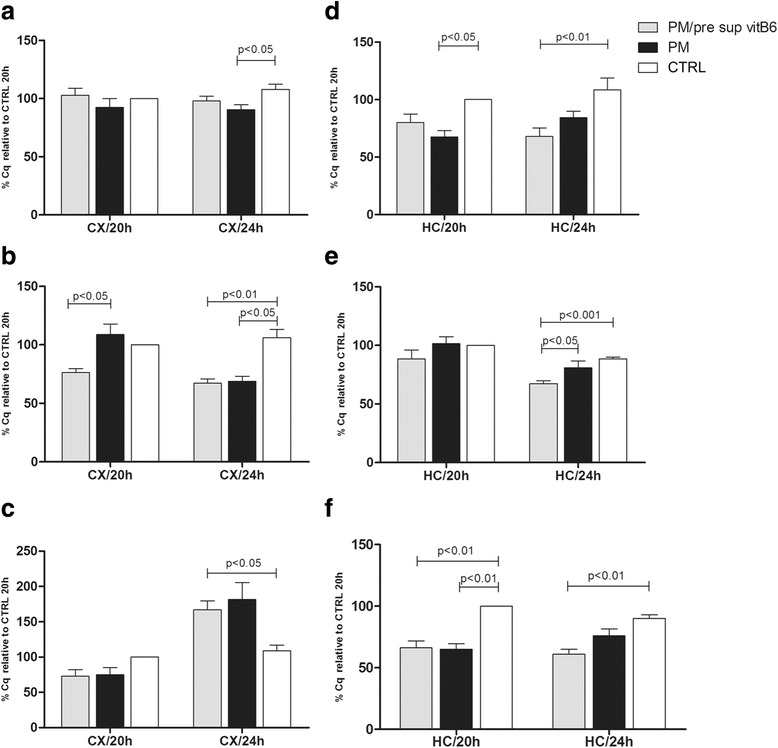
Effects of pre supplementation of vitamin B6 on APE1, AIF, and Casp-3 mRNA expression levels. Gene expression was determined in the CX (**a**, **b**, **c**) and HC (**d**, **e**, **f**) by real-time PCR and the fold change was calculated using the ΔCq method. Values were transformed to percentage using the control group at 20 h post infection as 100%. Each column data expresses the mean percentage (± SEM) relative to control 20 h. Comparisons between columns which show *p* < 0.05 were significant in Dunn’s post test. Groups: uninfected control animals (CTRL) 20 h (*n* = 5), uninfected control animals (CTRL) 24 h (*n* = 5), infected animals (PM) without vitB6 treatment 20 h p.i. (*n* = 5), infected animals (PM) without vitB6 treatment 24 h p.i. (*n* = 5), infected animals plus vitB6 treatment (PM/pre sup vitB6) 20 h p.i. (*n* = 8), infected animals plus vitB6 treatment (PM/pre sup vitB6) 24 h p.i. (*n* = 10)

The mRNA levels of APE1, AIF, and Casp-3 in the CX and HC at 20 and 24 h during PM and PM/pre sup vitB6 were determined. APE1 levels in CX/20 h did not show a significant difference among the various groups while CX/24 h showed lower levels of APE1 in the animals with PM compared to CTRL (Fig. 1a). AIF mRNA levels in the CX/20 h were significantly decreased in the PM/vitB6 group compared to those in the PM, while in the CX/24 h, a significant reduction in the AIF expression in the groups PM/vitB6 and PM was observed (Fig. 1b). Casp-3 did not show significant alterations in the CX/20 h, but was significantly higher compared to CTRL in the CX/24 h of PM/vitB6 group (Fig. 1c). Despite the vitB6 supplementation, infection in the animals resulted in significant reduction of APE1 and Casp-3 mRNA levels compared to CTRL animals in the HC/20 h tissue (Fig. 1d, f). All three studied genes were significantly reduced in the HC/24 h of PM/vitB6 animals compared to the CTRL group (Fig. 1d–f). Concomitantly, in the HC/24 h, lower AIF mRNA levels were noted in the animals from PM/vitB6 group compared to PM (Fig. 1e).

### Assessment of protein levels of APE1

**Fig. 2:**
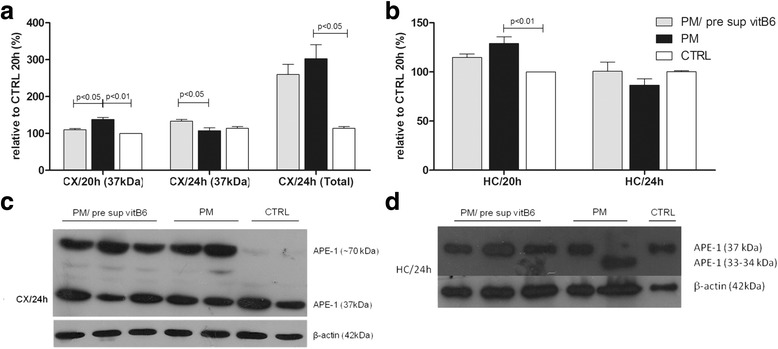
Western blotting (WB) analysis of the cortex (**a**, **b**) and hippocampus (**c**, **d**) tissues from Winstar rats infected with PM under vitB6 pre-supplementation treatment: **a** Significant reduction in the expression of APE1 is observed in the CX/20 h in the PM/pre sup vitB6 animals compared to the PM animals; **b** In the CX/24 h, WB from infected animals showed different sizes of APE1 (37 and 70 kDa) which resulted in a higher expression as compared to the control group as observed in **a**; **c** In HC/20 h, a significant increase in APE1 protein level was observed in the PM animals compared to the CTRL groups; **d** WB from the HC showed a cleaved form of APE1 in some infected animals which was not detected in the lysates from animals which received vitB6. β-actin was measured as the loading control and was used for data normalization. Data are reported as the mean percentage relative to control of each time point ± SEM. Groups for the CX: uninfected control animals (CTRL) 20 h (*n* = 5), uninfected control animals (CTRL) 24 h (*n* = 3), infected animals (PM) without vitB6 treatment 20 h p.i. (*n* = 5), infected animals (PM) without vitB6 treatment 24 h p.i. (*n* = 3), infected animals plus vitB6 treatment (PM/pre sup vitB6) 20 h p.i. (*n* = 9), and infected animals plus vitB6 treatment (PM/pre sup vitB6) 24 h p.i. (*n* = 3). Groups for the HC: uninfected control animals (CTRL) 20 h (*n* = 3), uninfected control animals (CTRL) 24 h (*n* = 3), infected animals (PM) without vitB6 treatment 20 h p.i. (*n* = 5), infected animals (PM) without vitB6 treatment 24 h p.i. (*n* = 3), infected animals plus vitB6 treatment (PM/pre sup vitB6) 20 h p.i. (*n* = 4), and infected animals plus vitB6 treatment (PM/pre sup vitB6) 24 h p.i. (*n* = 3)

Cortical APE1 expression showed higher levels during PM infection at both time points compared to CTRL animals (Fig. 2a). Comparing animals that received prior vitB6 supplementation and those without vitB6, APE1 protein showed lower levels in the CX/20 h, but an increase in APE1 (37 kDa) was observed in CX/24 h (Fig. 2a) in animals treated with vitB6. Surprisingly, a slower migrating form of APE1 was observed in the CX/24 h from animals with PM and PM/pre-sup vitb6; however, the same event was not observed in the CX/20 h. Consequently, these groups showed higher levels of total APE1 protein compared to CTRL animals (Fig. 2a, b). In the hippocampus tissue, PM infection caused a significant increase of APE1 protein at 20 h p.i. compared to CTRL animals (Fig. 2c). Although APE1 reduction has been noted in HC/20 h after vitB6 administration, it was not sufficient to promote significant changes in the DNA repair enzyme expression levels (Fig. 2c). Notably, complete cleavage of APE1 protein resulting in a smaller protein around 33–34 kDa was observed in the HC of some infected animals without vitB6 supplementation. However, this cleaved protein was not detected in the HC from animals supplemented with vitB6 or from the CTRL group (Fig. 2d).

### Vitamin B6 promotes lower glutamate levels and carbonyl content in the CX during PM

**Fig. 3:**
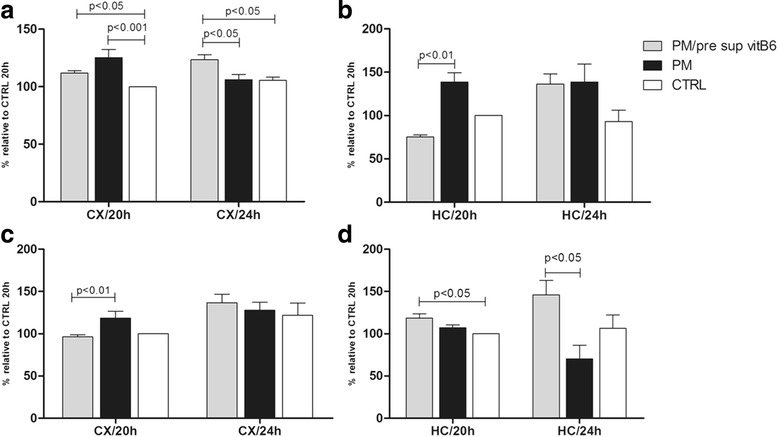
Levels of glutamate (**a**, **b**) and carbonyl content (**c**, **d**) in the CX and HC tissue from animals with PM after vitB6 supplementation. Glutamate level is significant increased during infection in both tissues and vitB6 has an effective influence up to 20 h p.i.. Reduction of carbonyl content were significant only in the CX/20 h in PM/pre sup vitB6. In contrast, in the HC, the vitB6 is associated to the highest level of carbonyls. Values were transformed to percentage using the control group at 20 h post infection as 100%. Each column data expresses the mean percentage (± SEM) relative to control 20 h from at least three or more animals. Comparison between columns which show *p* < 0.05 were significant in Dunn’s post test. Groups for the CX: uninfected control animals (CTRL) 20 h (*n* = 3), uninfected control animals (CTRL) 24 h (*n* = 5), infected animals (PM) without vitB6 treatment 20 h p.i. (*n* = 5), infected animals (PM) without vitB6 treatment 24 h p.i. (*n* = 5), infected animals plus vitB6 treatment (PM/pre sup vitB6) 20 h p.i. (*n* = 9), and infected animals plus vitB6 treatment (PM/pre sup vitB6) 24 h p.i. (*n* = 9). Groups for the HC: uninfected control animals (CTRL) 20 h (*n* = 3), uninfected control animals (CTRL) 24 h (*n* = 3), infected animals (PM) without vitB6 treatment 20 h p.i. (*n* = 3), infected animals (PM) without vitB6 treatment 24 h p.i. (*n* = 3), infected animals plus vitB6 treatment (PM/pre sup vitB6) 20 h p.i. (*n* = 9), and infected animals plus vitB6 treatment (PM/pre sup vitB6) 24 h p.i. (*n* = 4)

Glutamate, a major excitatory amino acid neurotransmitter, causes apoptotic neuronal cell death at high concentrations via increased oxidative stress and is a substrate of several vitB6-dependent enzymes. Analysis performed in the CX/20 h showed accumulation of glutamate levels during infection and its marginal but non-significant reduction after vitB6 supplementation. Nonetheless, the CX/24 h of infected animals supplemented with vitB6 showed significantly higher glutamate levels compared to the other groups (Fig. 3a). Glutamate levels were significantly reduced in the HC/20 h after vitB6 supplementation while no significant change was observed among groups in the HC/24 h (Fig. 3b). Corroborating the glutamate data, animals supplemented with vitB6 had lower concentrations of carbonylated proteins in the CX/20 h compared to animals without treatment, while no significant difference in the carbonyl content was noted in the CX/24 h among the various groups (Fig. 3c). Contrary to prior observations in the glutamate levels, the carbonyl content in HC/20 h and HC/24 h was significantly increased in the animals after vitB6 administration (Fig. 3d).

### Effect of an additional dose of vitB6 supplementation on APE1 expression levels in the PM cortex

**Fig. 4:**
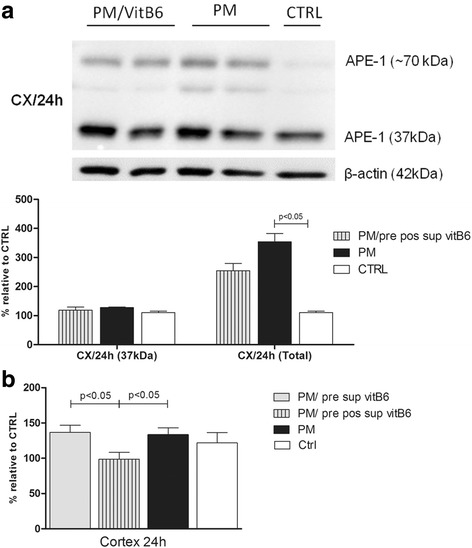
Effect of an additional dose of vitB6 on APE1 protein expression (**a**) and carbonyl content (**b**) in the CX/24 h. Western blot images reveal a reduction in the expression of different APE1 forms after vitB6 pre/post supplementation as indicated in the graph (**a**). Carbonyl content was significantly reduced after an extra dose of vitB6 (**b**). Each column from carbonyl data expresses the mean percentage (± SEM) relative to control CX/20 h (showed in Fig. 3c). Comparison between columns which show *p* < 0.05 were significant in Dunn’s post test. Groups: uninfected control animals (CTRL) (*n* = 3), infected animals (PM) without vitB6 treatment (*n* = 6), infected animals plus vitB6 treatment (PM/pre sup vitB6) (*n* = 9), and infected animals plus second dose of vitB6 treatment (PM/pre pos sup vitB6) (*n* = 5)

Since vitB6 had an enhanced effect in the cortex at 20 h p.i. compared to 24 h p.i. (Figs. 2a and 3a), the effect of an additional dose of vitB6 on the APE1 expression in the cortex was studied. Since vitB6 supplementation did not significantly influence the expression levels in the hippocampal tissue, it was not chosen for this study. A qualitative but statistically non-significant decrease in the total amount of APE1 protein was noted in the vitB6 group. The 70 kDa band of APE1 protein remained even after an extra dose of vitB6 (Fig. 4a). Additionally, carbonylated content was significantly reduced in the CX 24 h p.i. from animals pre pos supplemented with vitB6 (Fig. 4b).

## Discussion

The role of APE1 during oxidative stress response in the brain has been previously described [4, 5]. Over the last decade, APE1 has been associated with inflammation apart from its apurinic/apirimidinic endonuclease function [8]. In this work, our study demonstrated that APE1 expression can be modulated during PM according to the brain tissue, time of infection, and vitB6 supplementation.

Neuronal death in the CX during PM has been predominantly associated with necrosis [21]. However, our data suggest significant increase in AIF levels in the CX/20 h. In the previous studies using similar experimental model of PM, presence of AIF had been reported in the dentate gyrus of HC, where apoptosis is the predominant type of death [22, 23]. Nevertheless, a body of evidences has revealed an AIF-mediated programmed necrosis, which is initiated by the sequential activation of well-known proteins including PARP-1, calpain, and Bax [24, 25]. AIF translocation from the mitochondria to cytosol and nuclei has also been noted in primary cortical cells after glutamate induction [26]. Our data also showed significantly higher glutamate levels in the CX/20 h p.i. during PM compared to CTRL, which corroborate the AIF data. Since glutamate is known to activate *N*-methyl-*D*-aspartate (NMDA) receptor leading to influx of Ca^2+^, the excessive amount of ions in the cell promotes activation of calpain I and neuronal nitric oxide synthase (nNOS), thereby increasing the RNS/ROS levels.

DNA damage due to free radicals promotes PARP-1 overactivation and results in the energy depletion followed by AIF-mediated necrosis [24]. Interestingly, the amount of APE1 transcripts showed similar expression pattern compared to the two important proteins related to cell death, AIF, and Casp-3. Although, our data showed reduced levels of Casp-3 mRNA after PM, activity of this cysteine protease has been demonstrated mainly in the HC from PM animals [27]. Western blot analyses for the Casp-3 precursor protein (32 kDa) showed decreased immunoreactivity in the course of the disease, while an increase in the 17-kDa cleaved form of active Casp-3 was observed [27]. However, two microarray studies using similar experimental model of PM did not show significant upregulation of Casp-3 [16, 23], which can be the result of factors such as energy depletion [28] and nitrative/oxidative stress [29, 30]. In fact, neurons are susceptible to both energy depletion and oxidative stress. Therefore, the notable presence of APE1, AIF, and Casp-3 in the HC and CX may suggest an important tissue-dependent coordination among them during neuronal stress and may reinforce the idea of important mechanisms of cell death in the HC in addition to Casp-3-mediated mechanism. Collectively, we propose that AIF may play a more important role in the neuronal death compared to Casp-3.

Additionally, this work also showed elevated levels of APE1 protein during PM compared to CTRL and an intriguing post-translational modification (PTM) in the CX/24 h p.i., wherein a slower migrating form of APE1 (70 kDa) was observed in the infected animals with and without vitB6. Although the identity of this band has not been determined, we suggest a possible mechanism to address APE1 degradation since both mRNA and protein (37 kDa) levels of APE1 are lower in CX/24 h from PM animals. The protein ubiquitin E3 ligase MDM2, involved in the ubiquitination of p53 and responsible for its degradation, was previously demonstrated to interact directly with APE1 and promotes its ubiquitination, although the precise function of APE1 ubiquitination is not clear as yet [31]. In our work, elevated levels of APE1 and a truncated form of APE1 protein (34 kDa) was observed in the animals that did not receive vitB6 treatment. Conversely, vitB6 showed a protective effect in the hippocampus of the animals preventing the cleavage of APE1. The truncated form of APE1 is associated with apoptosis [32–34]. It has been demonstrated in human promyelocytic leukemia HL-60 cells that this truncated form of APE1 was involved in the apoptosis and DNA fragmentation, and its activation was mediated by Casp-3 [35]. Interestingly, apoptosis is the major type of cell death in the hippocampus during PM and is predominantly mediated via Casp-3 [27]. This observation suggests the possible connection between Casp-3 and APE1 during apoptotic cell death in the HC during PM.

The effect of vitB6 supplementation on the CX was mainly at the time point 20 h p.i.. The animals showed reduced levels of AIF, APE1, glutamate, and protein carbonyl at this time point. VitB6 is known to increase gamma-aminobutyric acid (GABA) levels, a major neurotransmitter acting in the inhibition of neurons, and may protect hippocampal CA1 pyramidal cells from ischemic damage [36]. Additionally, glutamate is an important precursor of GABA and glutamate decarboxylase (GAD) is the main enzyme specialized to synthesize GABA, which uses vitB6 as the cofactor [37]. Therefore, our data suggests a possible increase in the GAD activity and GABA concentrations although not experimentally validated. Consistent with this hypothesis, our study showed that animals treated with vitB6 had reduced levels of glutamate in both tissues at 20 h p.i., while the carbonyl content was reduced mainly in the CX at the same time point. Further, the chosen time points suggested a limited efficacy of this vitamin that was noted after the administration of an additional dose of vitB6. Animals that received two doses of vitB6 showed decreased APE1 protein expression and carbonyl content compared to those that received a single dose. Although reduced levels of hippocampal apoptosis have been reported in the prior study with additional supplementation of vitB6 at the same time point [16], it would be interesting to study the kinetics of vitB6 concentration to better understand the effects of vitB6 supplementation.

APE1 emerges as an intriguing candidate involved in the intricate network of cell death proteins once it responds directly to oxidative stress mediated by vitB6. Briefly, during PM, higher levels of glutamate induces activation of NMDA receptors promoting ROS production leading to DNA damage and, consequently, the requirement of DNA repair enzymes in the CX and cleavage of this protein in the HC. Changes in the permeability of the mitochondrial membrane promote the release of AIF and trigger apoptosis. Conversely, vitB6 supplementation reduces glutamate concentrations via GABA formation that block NMDA receptors and the following steps in the pathway. Hence, vitB6 promotes reduction of glutamate and oxidative damage and lowers the APE1 levels. Considering the interval of 18 to 24 h as the cell death hallmark during PM, we conclude that the maximum effect of vitB6 occurs at the beginning of the acute phase in CX.

## Conclusions

VitB6 pre-supplementation has a transient effect on the APE1 expression in the brain during PM. Therefore, reduced glutamate and carbonyl levels suggest that APE1 might be responding to ROS levels rather than to vitB6 itself although the precise mechanism remains to be uncovered. Consistent with this work, APE1 is emerging as a protein involved in the physiology of PM, which leaves some questions unanswered: Can the truncated APE1 form found in HC contribute to neuron death in the HC? Why does APE1 expression differ in the CX and HC? Besides the DNA repair activity, this protein is known to perform a variety of functions, which can be further exploited during PM. Altogether, vitB6 supplementation seems to show a possible beneficial effect majorly on the APE1 levels during PM which may be expanded to analyze other potential targets.

## Abbreviations

AIF: Apoptosis-inducing factor
APE1: Apurinic/apyrimidinic endonuclease 1
BER: Base excision repair
CA: Corrected absorbance
Casp-3: Caspase-3
CNS: Central nervous system
CSF: Cerebrospinal fluid
CX: cortex
DNPH: 2,4-Dinitrophenylhydrazine
GABA: Gamma-aminobutyric acid
GAD: Glutamate decarboxylase
GAPDH: Glyceraldehyde-3-phosphate dehydrogenase
HC: Hippocampus
NMDA: *N*-methyl-*D*-aspartate
p.i.: Post infection
PM: Pneumococcal meningitis
PTM: Post-translational modification
ROS: Reactive oxygen species
vitB6: vitamin B6
